# Advanced Materials for Energy Applications: From Fuels to Batteries and Beyond

**DOI:** 10.3390/molecules30071405

**Published:** 2025-03-21

**Authors:** Zhao Ding, Liangjuan Gao, Shicong Yang

**Affiliations:** 1 College of Materials Science and Engineering, National Engineering Research Center for Magnesium Alloys, Chongqing University, Chongqing 400044, China; 2 College of Materials Science and Engineering, Sichuan University, Chengdu 610065, China; lgao87@scu.edu.cn; 3 Faculty of Metallurgical and Energy Engineering, Kunming University of Science and Technology, Kunming 650093, China; shicongyang@kust.edu.cn

The unprecedented challenges of the 21st century energy landscape necessitate a paradigm shift in materials science and engineering [1,2,3,4]. The intricate interplay between rising global energy demands and environmental sustainability has catalyzed intensive research efforts across the entire energy value chain [5,6,7,8]. This Special Issue, “Advanced Materials for Energy Applications: From Fuels to Batteries and Beyond”, presents a carefully curated collection of cutting-edge research that exemplifies the transformative role of advanced materials in addressing these multifaceted challenges.

The ten papers featured in this issue collectively demonstrate the remarkable versatility and innovation in materials design, synthesis, and characterization. These contributions span multiple technological domains and can be systematically categorized into four complementary research directions: (1) advanced functional materials for environmental monitoring and remediation, (2) next-generation energy storage systems, (3) materials processing and industrial optimization, and (4) theoretical modeling coupled with advanced characterization techniques.

In the domain of environmental monitoring and remediation, two notable studies showcase innovative approaches to addressing heavy metal contamination. Liu et al. (contribution 1) developed a highly sensitive electrochemical sensing platform utilizing TiO_2_-modified biochar derived from spent coffee grounds. The hierarchical composite structure demonstrated remarkable sensitivity toward Pb(II) detection, achieving a detection limit of 0.6268 pM under optimized conditions. This work exemplifies the synergistic integration of waste valorization and advanced materials design. Complementing this research, Xu et al. (contribution 2) engineered sophisticated network polymer-modified superparamagnetic silica nanoparticles (MSNPs-CAAQ) for heavy metal remediation. Their systematic investigation revealed impressive maximum adsorption capacities of 324.7, 306.8, and 293.3 mg/g for Fe^3+^, Cu^2+^, and Cr^3+^ ions, respectively, with excellent recyclability maintained over multiple adsorption–desorption cycles.

Significant advances in energy storage technologies are highlighted through innovative materials development and systematic performance enhancement studies. Wang et al. (contribution 3) pioneered the development of a bio-derived separator for sodium metal batteries, utilizing natural silkworm cocoon membrane. The innovative separator design, featuring intrinsic cationic functional groups, demonstrated superior Na-ion transport kinetics and enhanced electrochemical performance, achieving an initial capacity of 79.3 mAh g^−1^ at 10C with 93.6% retention after 1000 cycles. Abdisattar et al. (contribution 4) conducted a comprehensive investigation into biomass-derived activated carbons for supercapacitor applications, establishing critical structure–property relationships between lignocellulosic precursor composition and electrochemical performance. Their findings revealed that wheat bran-derived electrodes exhibited optimal performance characteristics, attributed to their unique hierarchical pore structure and surface chemistry. Wang et al. (contribution 5) further contributed to this domain through a comprehensive review of strategies for enhancing LiFePO_4_ cathode conductivity, while Wang et al. (contribution 6) provided an in-depth comparative analysis of solid electrolyte interface formation mechanisms in Li and Mg batteries.

In the realm of materials processing and industrial optimization, several significant contributions advance our understanding of crucial manufacturing processes. Liu et al. (contribution 7) conducted a detailed investigation of the sintering behavior in molybdenite concentrate oxidation, elucidating the complex relationships between processing parameters and product characteristics. Their systematic study revealed the critical influence of temperature and potassium content on sintering mechanisms, providing valuable insights for process optimization. Additionally, Zhu et al. (contribution 8) developed an innovative flotation technique for high-purity silicon recovery from diamond wire saw silicon slurry, achieving remarkable recovery rates of 98.2% under optimized conditions, thereby addressing both resource recovery and environmental sustainability challenges.

The theoretical understanding and characterization of advanced materials have been significantly enhanced through sophisticated studies employing cutting-edge analytical techniques and computational methods. Zhou et al. (contribution 9) employed density functional theory calculations to elucidate the complex interfacial phenomena in Mg/Ti systems, providing fundamental insight into the role of various alloying elements. Their computational analysis revealed that Gd exhibits the most favorable segregation behavior with a segregation energy of −5.83 eV, offering valuable guidance for the rational design of magnesium-based composites. Xu et al. (contribution 10) presented an authoritative review of characterization methodologies in metal-based solid-state hydrogen storage, comprehensively analyzing both conventional and emerging analytical techniques, particularly emphasizing the crucial role of in situ and operando characterization in understanding hydrogen–material interactions at atomic and molecular levels.

Looking ahead, several emerging trends and research directions are evident from these contributions. The integration of artificial intelligence with materials design, the development of multifunctional materials for energy applications, and the optimization of sustainable production methods represent particularly promising avenues for future research. The continued evolution of in situ characterization techniques and theoretical modeling capabilities will be crucial in accelerating materials discovery and optimization. As we progress toward a more sustainable and resilient energy future, the strategic development of advanced materials remains paramount. The papers in this Special Issue demonstrate the remarkable potential of innovative materials solutions in addressing global energy challenges, while simultaneously highlighting the importance of interdisciplinary approaches and sustainable methodologies in materials research and development.
